# Synergistic Effects of Serum Uric Acid and Cardiometabolic Risk Factors on Early Stage Atherosclerosis: The Cardiometabolic Risk in Chinese Study

**DOI:** 10.1371/journal.pone.0051101

**Published:** 2012-12-17

**Authors:** Jun Liang, Yanping Li, Na Zhou, Fei Teng, Jing Zhao, Caiyan Zou, Lu Qi

**Affiliations:** 1 Department of Endocrinology, the Central Hospital of Xuzhou, Xuzhou Clinical School of Xuzhou Medical College, Affiliated Hospital of Southeast University, Xuzhou, Jiangsu, People's Republic of China; 2 Department of Nutrition, Harvard School of Public Health, Boston, Massachusetts, United States of America; 3 Channing Laboratory, Department of Medicine, Brigham and Women's Hospital and Harvard Medical School, Boston, Massachusetts, United States of America; 4 Oriental People's Hospital of Xuzhou, Xuzhou, Jiangsu, People's Republic of China; University of Virginia Health System, United States of America

## Abstract

**Objective:**

To comprehensively examine the associations of serum uric acid (SUA) with central and peripheral arterial stiffness in Chinese adults, and particularly assess the interactions between SUA and other cardiometabolic risk factors.

**Methods:**

The study included 3,772 Chinese men and women with carotid radial pulse wave velocity (crPWV), carotid femoral PWV (cfPWV), carotid artery dorsalis pedis PWV (cdPWV) and SUA measured.

**Results:**

After adjustment for age, sex, and BMI, the levels of SUA were significantly associated with increasing trend of cfPWV, crPWV and cdPWV (*P* for trend <0.0001). Further adjustment for heart rate (HR), blood pressure (BP) and lipids attenuated the associations with crPWV and cdPWV to be non-significant (*P* = 0.1, *P* = 0.099 respectively), but the association between SUV and cfPWV remained significant (P = 0.004). We found significant interactions between SUA and HR or BP in relation to cfPWV (*P* for interaction = 0.03, 0.003 respectively). The associations between SUA and cfPWV were more evident among individuals with higher HR or normal BP than those with lower HR or hypertension.

**Conclusions:**

SUA was associated with elevated aortic arterial stiffness in Chinese adults, independent of conventional cardiovascular risk factors. BP and HR might modify the deleterious effects of SUA.

## Introduction

Serum uric acid (SUA) is a final enzymatic product of purine metabolism in humans. In clinical and epidemiological studies, SUA has been related to the risk of hypertension [1]–[3], atherosclerosis [4] and cardiovascular diseases (CVDs) [5], [6]. The adverse effects of SUA may occur at early stage of atherosclerosis [7], [8]. Pulse wave velocity (PWV) is a gold standard for assessing arterial stiffness and widely used indicator for early atherosclerosis [9], [10], and PWVs at different sites may reflect the atherosclerotic alterations at central (e.g. cfPWV) or peripheral arteries (e.g. cdPWV and crPWV). Few studies have comprehensively compared the effects of circulating SUA on these various measures.

In addition, it has been documented that both blood SUA levels and arterial stiffness are tightly related to other cardiometabolic risk factors such as high heart rate (HR) or blood pressure (BP) [2], [3], [11], [12]. However, few studies have examined the potential interactions between SUA and those factors. It remains to be determined whether other cardiometabolic risk factors may modify the cardiovascular effects of SUA.

In the present study of a large sample of Chinese adults, we sought to comprehensively evaluate the effects of SUA on peripheral and central arterial stiffness. We particularly assessed whether blood pressure, heart rate, and other cardiometabolic risk factors may modify the relation between SUA and arterial stiffness.

## Methods

### 1. Study Population

In the Cardiometabolic Risk in Chinese (CRC) Study, we performed a community-based health examination survey for subjects (18–93 y) who were randomly selected from residents living in the urban area of Xuzhou, China, in 2009. Written consents were obtained from all the participants. The study was reviewed and approved by the ethics committee of the Central Hospital of Xuzhou, Affiliated Hospital of Medical School of Southeast University, China. For the present study, we included adult men and women (≥18 y) who were successfully measured for PWVs, BP, Body mass index (BMI), HR, SUA and other metabolic markers. The exclusion criteria included the history of vascular disease, diabetes mellitus, or hyperlipidemia which was being treated with medication, and renal failure (GFR reduced to 10∼20% and serum creatinine elevated to 451∼707 umol/L) [13]. In addition, we excluded people who did not undergo PWV determination or omitted blood sampling. In total 3,772 men and women were included in the final analyses. There was not significant difference in basic characteristics such as age, education, and anthropometrics between individuals included in the analyses and those who were excluded.

### 2. Assessment of PWVs

All measurements were performed in a quiet room with controlled ambient temperature. The cfPWV was measured in the supine position after 5 min of bed rest using an automatic waveform analyzer (Complior System, Artech-Medical corp. French), the pulse wave of the carotid and femoral arteries was analyzed, estimating the delay with respect to the ECG wave and calculating the PWV. cdPWV and crPWV were obtained in a similar way, with the pulse wave being measured simultaneously in the right radial, dorsum of foot and right carotid arteries. 16 consecutive electrocardiogram gated waveforms were obtained and removed the three maximum and three minimum. For analysis, we averaged 10 waveforms. PWV was based on the distance/time ratio (meters/second), was calculated as the path length divided by the transit time and expressed as m/s [14].

### 3. Assessment of Biomarkers and covariates

All the participants were measured biomarkers. Venous blood sample was drawn from all subjects after an overnight fast (8–12 h). After blood was drawn, samples were allowed to clot at room temperature for 1–3 h and serum was separated. Immediately after clotting, serum was separated by centrifugation for 15 min at 3000 r.p.m. Fasting blood samples were collected for measurement of glucose, SUA, total cholesterol (TC), triglyceride (TG), high-density lipoprotein cholesterol (HDL-C) and low-density lipoprotein cholesterol (LDL-C). All biochemical assays were determined enzymatic ally on an auto analyzer (Type 7600, Hitachi Ltd, and Tokyo, Japan). Height was measured to the nearest 0.5 cm without shoes and body weight was measured to the nearest 100 grams without shoes. BMI was calculated as weight (in kilograms) divided by height (in meters) squared. BP was measured after the subject had rested for at least 5 minutes with a mercury manometer by doctors. The mean arterial pressure (MAP) was calculated as 2/3(Diastolic blood pressure,DBP)+1/3(Systolic blood pressure,SBP). Three measurements, 60 seconds apart, were taken. The mean of the three measurements was used for analysis. Information of education level, income smoking status and alcohol consumption was collected using a questionnaire.

### 4. Statistical Analysis

The relations between SUA levels (in quartiles) and PWVs were examined using general linear regression models, adjusting for covariates including age, sex, BMI, HR, fasting glucose, lipid profiles and BP. SUA and TG levels were log-transformed to improve normal distribution before analysis. The interactions between SUA and other cardiometabolic risk factors were assessed by introduction of cross-product term in the regression models. All reported P values are two tailed. Variables with P values of <0.05 were considered statistically significant. Data management and statistical analysis were conducted using SAS statistical software (version 9.1; SAS Institute, Inc., Cary, NC, USA).

## Results

### 1. The characteristics of the study participants by SUA levels

The average age of study population was 45.4 years and was represented by 63.3% of men. **Table 1** shows the characteristics of the study participants according to SUA levels (in quartiles). BMI, waist circumference, BP, glucose, insulin, TG and LDL-C showed statistically significant differences between SUA groups, with an increasing trend as the concentration of SUA increased, except for HDL-C that showed a decreasing trend.

**Table 1:** Characteristics of the participants by serum uric acid (SUA) levels.

	SUA in quartiles(umol/L)				*P*
Variables	Q1 (≤238,N = 941)	Q2 (238–294.4, N = 943)	Q3 (294.4–348.9, N = 946)	Q4 (≥348.9,N = 942)	for trend
cfPWV(m/s)	9.83±0.05	10.39±0.06	10.75±0.06	11.0±0.06	0.009
cdPWV(m/s)	8.87±0.05	9.56±0.05	9.85±0.06	10.1±0.05	0.23
cr PWV(m/s)	9.59±0.05	10.27±0.05	10.68±0.06	11.0±0.06	0.003
Age, years	45.2±12.1	44.8±11.4	45.1±11.7	45.7±12.4	0.33
Men, %	63.3	63.3	63.3	63.4	1.00
Body mass index, kg/m^2^	23.5±0.1	24.1±0.1	24.4±0.1	25.8±0.1	<0.0001
Waist circumference, cm	83.2±0.3	84.4±0.3	85.7±0.3	89.1±0.3	<0.0001
Systolic blood pressure, mmHg	121.2±0.5	122.5±0.5	123.6±0.5	127.0±0.5	<0.0001
Diastolic blood pressure, mmHg	77.3±0.4	78.0±0.4	79.8±0.4	82.1±0.4	<0.0001
Fasting glucose, mmol/L	4.8±0.04	5.0±0.04	5.1±0.04	5.2±0.04	0.02
Fasting insulin (IU/ml)	7.5±0.14	8.4±0.15	9.0±0.17	11.2±0.24	<0.0001
2 h OGTT, mmol/L	7.48±0.10	7.15±0.10	7.29±0.10	7.72±0.11	0.03
Triglyceride, mmol/L	1.26±0.05	1.48±0.05	1.65±0.05	2.18±0.05	<0.0001
LDL-C (mmol/l)	2.8±0.03	2.9±0.03	3.0±0.03	3.1±0.03	<0.0001
HDL-C, mmol/L	1.30±0.01	1.27±0.01	1.25±0.01	1.20±0.01	<0.0001

Abbreviations: OGTT, oral glucose tolerance test; HDL-C, high density lipoprotein cholesterol; LDL-C, low-density lipoprotein cholesterol. Data are age and sex adjusted mean ± standard error.

Linear regression model was used to test trend for continuous variables; χ^2^ test was used for the categorical variables.

### 2. Association between SUA and markers of central and peripheral arterial stiffness

**Table 2** displays the associations of PWVs with SUA in quartile. After adjustment for age, sex and BMI, the levels of SUA were significantly associated with an increasing trend of cfPWV, crPWV and cdPWV in a dose-dependent pattern (*P* for trend <0.0001). Further adjustment for HR, fasting glucose and lipids did not significantly change the associations. However, further addition of blood pressure in the models attenuated the associations with crPWV and cdPWV to be not significant (*P* = 0.1, *P* = 0.099 respectively); while the association between SUV and cfPWV remained significant (P = 0.004). Additional adjustment of education level, income, smoking and alcohol consumption did not appreciably change the results.

**Table 2:** Associations of SUA with central and peripheral arterial stiffness.

	SUA in quartiles				*P*
Models	Q1	Q2	Q3	Q4	for trend
cf_PWV					
Crude	10.39 (0.06)	10.44 (0.06)	10.62 (0.06)	10.82 (0.06)	<.0001
Age and sex adjusted	10.39 (0.05)	10.46 (0.05)	10.63 (0.05)	10.79 (0.05)	<.0001
Further adjusted for BMI	10.41 (0.06)	10.47 (0.05)	10.63 (0.05)	10.80 (0.06)	<.0001
Further adjusted for fasting glucose, lipid profiles and heart rate	10.48 (0.06)	10.53 (0.06)	10.63 (0.06)	10.76 (0.06)	0.0006
Further adjusted for blood pressure	10.48 (0.05)	10.51 (0.05)	10.65 (0.05)	10.72 (0.05)	0.004
cd_PWV					
Crude	9.40 (0.05)	9.50 (0.05)	9.64 (0.05)	9.78 (0.05)	<.0001
Age and sex adjusted	9.40 (0.05)	9.51 (0.05)	9.64 (0.05)	9.77 (0.05)	<.0001
Further adjusted for BMI	9.46 (0.05)	9.52 (0.05)	9.64 (0.05)	9.71 (0.05)	<.0001
Further adjusted for fasting glucose, lipid profiles and heart rate	9.54 (0.05)	9.54 (0.05)	9.62 (0.05)	9.68 (0.05)	0.03
Further adjusted for blood pressure	9.56 (0.05)	9.54 (0.05)	9.62 (0.05)	9.65 (0.05)	0.099
cr_PWV					
Crude	10.12 (0.05)	10.29 (0.05)	10.40 (0.05)	10.39 (0.05)	<.0001
Age and sex adjusted	10.12 (0.05)	10.28 (0.05)	10.40 (0.05)	10.39 (0.05)	<.0001
Further adjusted for BMI	10.13 (0.05)	10.28 (0.05)	10.40 (0.05)	10.39 (0.05)	<.0001
Further adjusted for fasting glucose, lipid profiles and heart rate	10.20 (0.05)	10.33 (0.05)	10.40 (0.05)	10.38 (0.05)	0.014
Further adjusted for blood pressure	10.23 (0.05)	10.34 (0.05)	10.38 (0.05)	10.35 (0.05)	0.10

PWVs are presented as mean (standard error).

### 3. Stratified associations by cardiometabolic risk factors

We found significant interaction between age and SUA level in relation to cfPWV (P for interaction <0.001). (**Table 3**) The associations were significant in groups of 40 to 59 y (*P* = 0.03) and ≥60 y (P = 0.02), but not significant among those of <40 y (*P* = 0.18). There was no significant interactions of SUA with sex and BMI in relation to cfPWV.

**Table 3:** Stratified associations between SUA and cfPWV by sex, age and BMI.

	SUA in quartiles				P for	P for
	Q1	Q2	Q3	Q4	trend	interaction
Age						
<40 y; N = 1070	10.00 (0.09)	9.95 (0.08)	10.07 (0.08)	10.13 (0.08)	0.18	<0.001
40 to 59 y; N = 2347	10.41 (0.06)	10.44 (0.06)	10.53 (0.06)	10.57 (0.06)	0.03	
≥60 y; N = 355	12.52 (0.33)	13.00 (0.35)	13.56 (0.32)	13.54 (0.31)	0.02	
Sex						
Women, N = 1283	9.79 (0.08)	9.79 (0.08)	9.96 (0.08)	10.0 (0.08)	0.04	0.65
Men, N = 2389	10.86 (0.07)	10.91 (0.07)	11.03 (0.07)	11.09 (0.07)	0.01	
BMI						
<23 kg/m2; N = 1350	10.07 (0.08)	10.44 (0.08)	10.39 (0.09)	10.40 (0.12)	0.008	0.53
23 to 24.9 kg/m^2^; N = 895	10.59 (0.11)	10.37 (0.10)	10.62 (0.10)	10.56 (0.11)	0.70	
≥25 kg/m^2^; N = 1527	10.92 (0.10)	10.62 (0.09)	10.89 (0.08)	10.98 (0.07)	0.11	

Analyses were adjusted for age, sex, BMI, total cholesterol, triglyceride, HDL-C, LDL-C, blood pressure, heart rate and fasting glucose but not the strata variable. cfPWV is presented as mean (standard error).

**Figure 1:**
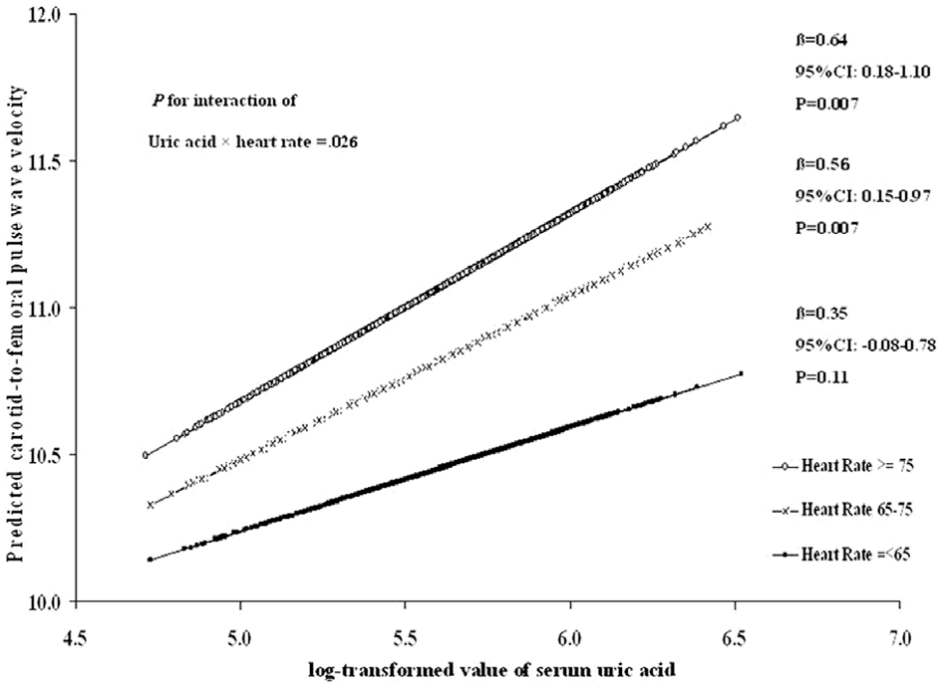
Interaction between SUA and HR in relation to cfPWV. The predicted cfPWV by log-transformed SUA in different HR categories: ≤65, 65–75 and ≥75 bpm are presented. Analysis was adjusted for age, sex, BMI, fasting glucose, lipids and BP.

**Figure 2:**
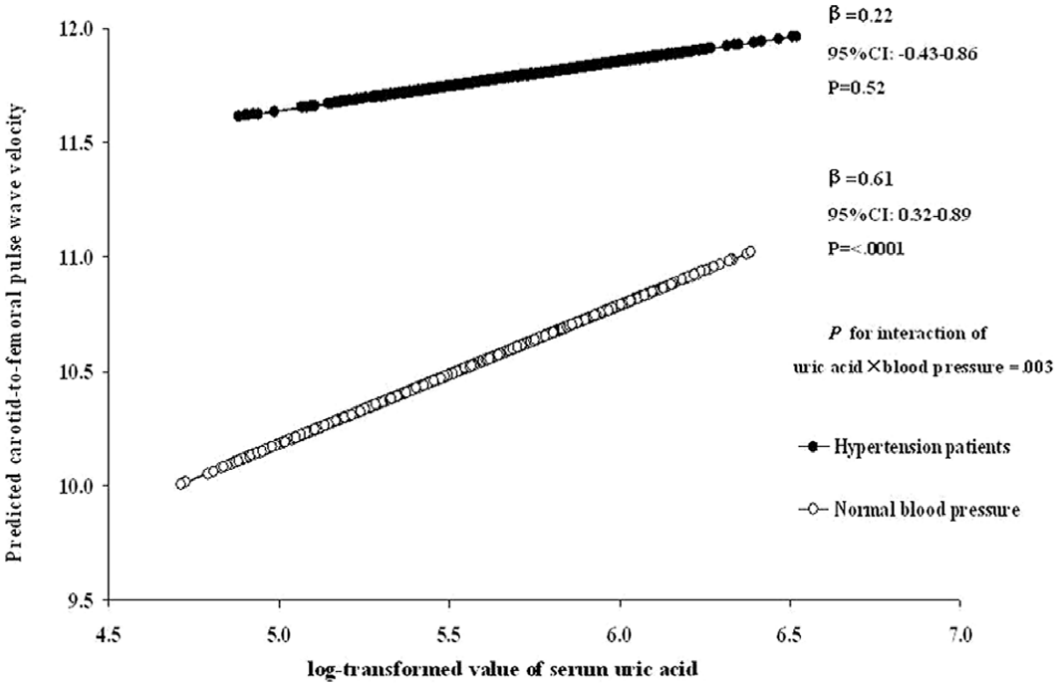
Interaction between SUA and hypertension in relation to cfPWV. The predicted cfPWV by log-transformed SUA in individuals with normal blood pressure and hypertension are presented. Analysis was adjusted for age, sex, BMI, fasting glucose, lipids and HR.

We also examined the associations between SUA and cfPWV in different HR categories: ≤65, 65–75 and ≥75 bpm (**Figure 1**). After adjustment for age, sex, BMI, fasting glucose, lipids and BP, we found that the association between SUA and cfPWV was different in three HR groups (P for interaction = 0.03). The associations were stronger in individuals with HR of 65–75 bpm (P = 0.007) and ≥75 bpm (P = 0.007) than those with HR<65 bpm (P = 0.11). In addition, we found significant interaction between hypertension status and SUA level in relation to cfPWV, adjusted for age, sex, BMI, fasting glucose, lipids and HR (P for interaction = 0.003). The associations between SUA and cfPWV were significant (P<0.0001) among those with normal blood pressure, but not significant among those with hypertension (**Figure 2**).

## Discussion

In the present study of a large sample of Chinese adults, we found that SUA levels were significantly related to central arterial stiffness independent of conventional risk factors, such as sex, BMI, lipids, glucose metabolism. SUA levels were not associated with peripheral arterial stiffness, measured by crPWV and cdPWV. Moreover, we found significant interactions between SUA and HR or BP in relation to cfPWV.

Several previous studies have shown that hyperuricemia was associated with cardiovascular disease [15], [16], and the detrimental effects of high SUA might occur at early stage of atheroclerosis [7], [8]. In a recent cross-sectional study, it was found that SUA was independently related to brachial ankle PWV (baPWV) in She ethnic minority of China [17]. However, it is not clear whether SUA levels specifically affect central or peripheral arterial stiffness because baPWV may be influenced by both sites [18]. Our results indicate that high SUA more likely affect central arterial stiffness; while its effects on peripheral arterial stiffness are modest. Notably, in the Framingham Heart Study, one standard deviation (SD) increment in arterial stiffness, as measured by cfPWV, was associated with a 48% increase in arterial disease risk, independently of conventional risk factors [19].

Intriguingly, we found the association between SUA and cfPWV was stronger among adults with higher HR (≥65). Several cohort studies have demonstrated that increased HR at rest is a significant risk factor for CVD and is a marker of new onset of atherosclerosis even in apparently healthy individuals [20], [21]. Evidence has also shown that elevated HR is directly associated with risk of developing hypertension and metabolic syndrome, and is a potent predictor of cardiovascular mortality [22]. A recent prospective study found a synergistic role of high baseline HR and changes in HR during the follow-up period in accelerating increases of baPWV [12]. These findings as well as our result suggest that high SUA and high HR may synergistically affect central arterial stiffness.

Another interesting finding is that, the adverse effects of SUA on central arterial stiffness appeared more evident in people with normal blood pressure than those with hypertension. Notably, on average cfPWV was much higher and its variance was smaller in individuals with hypertension than those with normal blood pressure (Figure 2). Therefore, it is not surprising a null association was observed between SUA and cfPWV in hypertensive patients. Recent clinical evidence and experimental studies showed that SUA levels contribute to incident hypertension and prehypertention [2], [3], [23], [24]. One prospective study showed that inflammatory and adiponectin-mediated proatherogenic activation are interrelated and interact leading to a significant increase of arterial stiffness in essential hypertensive patients [25]. Our findings suggest that high SUA levels may play a more important role in arterial stiffness before the development of hypertension. This finding might have important clinical implications for prevention and intervention of cardiovascular risk at early stage.

Our data showed a stronger effect of SUA on central arterial stiffness in older population. Shen et al. reported that cfPWV increased at the early stage of carotid artery atherosclerosis in elderly patients, compared to younger subjects [26]. Elevated SUA experimentally stimulates renal vasoconstriction and activation of the renin-angiotensin system. Senior age is associated with activation of the renin–angiotensin system and with renal vasoconstriction [27]. Also increased SUA levels are accompanied by a state of pronounced inflammatory activation and hypoadiponectinemia that significantly impairs the arterial stiffness accelerating the vascular ageing process [28]. Our results suggest that older individuals may be more sensitive to the detrimental effects of high SUA on atherosclerosis.

The sample size of the present study is large, which ensures sufficient power to detect the moderate effects of SUA on arterial stiffness and interactions with other cardiometabolic risk factors. However, several limitations of this study warrant consideration. Our study is cross-sectional in design. Therefore, a causal relation between SUA and arterial stiffness could not be derived. We have carefully adjusted for the potential confounding in the analyses. However, in our study samples, we did not collection information of dietary intake and the lifestyle information is not incomplete. Therefore, it is still possible the residual confounding of these unmeasured variables might influence the associations. In addition, the study was performed in a Chinese population. Further studies in other populations of different ethnicities are warranted to verify our findings.

## Conclusion

In conclusion, in Chinese adults we found that SUA was associated with elevated aortic arterial stiffness, independent of conventional cardiovascular risk factors. BP and HR might modify the deleterious effects of SUA. Our data lend support of the role of SUA in development of cardiovascular disease at early stage; and suggest to jointly consider the interactions of SUA with other risk factors in prevention of heart disease.
